# The global prevalence of and risk factors for fear of falling among older adults: a systematic review and meta-analysis

**DOI:** 10.1186/s12877-024-04882-w

**Published:** 2024-04-05

**Authors:** Wanhong Xiong, Dan Wang, Wei Ren, Xinyi Liu, Renhui Wen, Yu Luo

**Affiliations:** 1 School of Nursing, Third Military University / Army Medical University, No. 30 Gaotanyan Street, Shapingba District, Chongqing, P.R. China; 2 Department of Oncology, Zigong First People’s Hospital, Zigong, Sichuan China

**Keywords:** Fear of falling, Accidental Falls, Older adults, Geriatric nursing, Psychological nursing

## Abstract

**Background:**

As a common psychological problem among older adults, fear of falling was found to have a wide range prevalence in different studies. However, the global prevalence of it was unknown and a lack of the large sample confirmed its risk factors.

**Objectives:**

To report the global prevalence of fear of falling and to explore its risk factors among older adults for further developing precise interventions to systematically manage FOF.

**Design:**

A systematic review and meta-analysis was conducted by PRISMA guidelines.

**Methods:**

Searches were conducted in PubMed, Web of Science, EMBASE, the Cochrane Library and the manual search in August 20, 2022, updated to September 2, 2023. Observational studies published in English were included and two researchers independently screened and extracted the data. Fixed or random effects mode was used to estimate the pooled prevalence of and risk factors for fear of falling. Heterogeneity resources were analyzed by subgroup and sensitivity analysis. Publication bias was assessed through funnel plots, Egger’s test and Begg’s test.

**Results:**

A total of the 153 studies with 200,033 participants from 38 countries worldwide were identified. The global prevalence of fear of falling was 49.60%, ranging from 6.96–90.34%. Subgroup analysis found the estimates pooled prevalence of it was higher in developing countries (53.40%) than in developed countries (46.7%), and higher in patients (52.20%) than in community residents (48.40%). In addition, twenty-eight risk factors were found a significant associations with fear of falling, mainly including demographic characteristics, physical function, chronic diseases and mental problems.

**Conclusion:**

The global prevalence of FOF was high, especially in developing countries and in patients. Demographic characteristics, Physical function, chronic diseases and mental problems were a significant association with FOF. Policy-makers, health care providers and government officials should comprehensively evaluate these risk factors and formulate precise intervention measures to reduce FOF.

**Trial registration:**

The study was registered in the International Database of Prospectively Registered Systematic Reviews (PROSPERO): CRD42022358031.

**Supplementary Information:**

The online version contains supplementary material available at 10.1186/s12877-024-04882-w.

## Background

Falls have emerged as a major health issues, and health care is paying increasing attention to them as the primary cause of illness and early mortality among older adults. At least one in three older adults experience a fall each year, and fear of falling (FOF) causes more falls [1]. Negative fall-related consequences, such as fear of falling, increase the likelihood of disability and declining quality of life in relation to health [2]. FOF has been defined as the low perceived self-efficacy at avoiding falls in daily activities [3]. FOF is prevalent in older adults and may lead to several negative health outcomes such as changing gait [4], restricting daily activities [5], developing depression [5], increasing the risk of falls [6], and negatively impacting quality of life [2]. Earlier studies revealed that the 8-year mortality rate was nearly 14% greater among older adults with FOF than among those without FOF, and 16% greater among older adults with limited activities than among those without restricted activities [7]. In particular, avoiding certain situations can lead to fear of falling, which exacerbates poor physical health, balance concerns, and social isolation [8].

Fear of falling is common among older adults regardless of they have a history of falls. A study showed that the prevalence of FOF ranged from 3 to 85% among community-dwelling older adults with a history of falls [9]. However, different individuals and those from other nations reported the prevalence of FOF in different ways. FOF also occurs frequently among hospitalized patients [10, 11], especially among those who have undergone total knee arthroplasty [12], who have a hip fracture [13], and who have diabetic neuropathy [14]. Similarly, more than 50% of people with Parkinson’s disease had high levels of FOF, while close to 30% had moderate levels [15]. Previous studies demonstrated that there was significant variation in the incidence of FOF among older adults living in communities of different nationalities., ranging from 9.26% to 83.33% in the USA [16, 17], from 22.31% to 86.46% in Japan [18, 19], from 38.84% to 96.70% in Korea [20, 21], from 37.03% to 51.38% in Spain [22, 23], and from 44.56% to 86.71% in Turkey [24, 25]. At present, various tools are available to assess FOF, including the single question “Are you afraid of falling?”, the Falls Efficacy Scale (FES), the Falls Efficacy Scale-International (FES-I), the Short Falls Efficacy Scale International (SFES-I), the Modified fall efficacy scale(MFES) and the Activities-Specific Balance Confidence Scale (ABC). Moreover, earlier studies showed that similar results were obtained utilizing a single-question tool and various structured questionnaire instruments [26].

Age, female sex, balance, living alone, chronic illnesses, and psychiatric issues were all risk factors for FOF. People with neurological disease and a history of falls in the previous 6 months were more than twice as likely to have FOF, while those with depression were more than six times more likely to have FOF [27]. Compared with nonfrail older adults, those with a frail physical condition and lack of daily activity had a greater than three times greater risk of experiencing FOF, and both female sex and depression found to be independent predictors of FOF [28]. A systematic review revealed that FOF in stroke patients was closely correlated with female sex, impaired balance ability, decreased mobility, a history of falls and walking aids, and decreased weight, which may present more challenges for impaired balance ability and excessive safety awareness of life circumstances and daily activities, increasing the risk of FOF and leading to the development of psychological stress [29]. Evidence has shown that gait variability, such as slowing gait speed, shorter strides, and widening strides, is similarly associated with FOF; however there are no conclusive findings from brain imaging to date [4]. Cognitive impairment, which was confirmed to be a predictor of having a significant effect on a high level of FOF, could increase the risk of accepting high FOF by three times in the older adults with low and moderate levels of social support caused by limited social activity, lack of family support and an aging-unfriendly environment [30]. Additionally, chemotherapy might make cancer patients more feeble and reduce their postural and limb stability because taxanes and platinum drugs harm muscle and peripheral nerve tissue, which reduces the efficacy of falls [31].

In conclusion, FOF is complicated by people’s physical, psychological, and social support systems, and its occurrence varies widely among studies involving different subjects, tools, and nations. Notably, the global prevalence of FOF is currently unknown. Therefore, this study includes a systematic review and meta-analysis to address the limitations of previous studies by estimating the global prevalence of FOF among older adults and fully exploring its risk factors for further developing precise interventions to systematically manage FOF.

## Methods

### Design

This systematic review and meta-analysis was conducted according to the Reporting Items for Systematic Review and Meta-Analysis (PRISMA) guidelines and was registered in the International Database of Prospectively Registered Systematic Reviews (ROSPERO: CRD42022358031).

### Search methods

A systematic search of the literature was performed in PubMed, Web of Science, EMBASE, and the Cochrane Library from the inception of the database until August 20, 2022, and updated to September 2, 2023. The search strategies were developed using a combination of MeSH terms and free words with Boolean operators. The details were as follows: (“Aged”[Mesh] OR “older” OR “older adult” OR “older” OR “elderly” OR “the aged”) AND (“fear of falling”) AND (“influence factors” OR “risk factors”) (see PubMed search strategies in Supplementary Material 1) and manually searched methods were used to check potential studies.

### Inclusion and/or exclusion criteria

After removing duplicate studies, two reviewers independently reviewed studies based on the title and abstract and subsequently screened the literature based on full-text via the inclusion and exclusion criteria. The inclusion criteria for the studies were as follows: 1) participants were at least 60 years old; 2) the prevalence or risk factors for FOF were reported; 3) the study design was observational study, including cohort, case-control and cross-sectional studies; and 4) the study was published in the English language. The exclusion criteria were as follows: 1) full-text could not be obtained, or 2)incomplete or erroneous data.

### Data extraction

Data were extracted independently by two researchers with the following variables: first author name, publication year, country, type of study instrument, subject, age, female ratio, sample size, prevalence of FOF, quality of studies and risk factors for FOF. If FOF was assessed using kinds of different instruments, the prevalence of FOF was extracted according to the results of the eligible studies. All disagreements were resolved by discussion between two researchers, and a third researcher was consulted if needed.

### Quality assessment

Two researchers independently assessed the possibility of bias, and disagreements were settled by discussion or consultation with a third researcher. The quality of the case-control and cohort studies was assessed by using the Newcastle–Ottawa Scale [32], which has 8 items and a total score ranging from 0 to 9, and scores ranging from 0 to 3, 4 to 6 and 7 to 9 indicated low, medium and high quality, respectively. The quality of cross-sectional studies was assessed by using the instrument Agency for Healthcare Research and Quality (AHRQ) [33] with a total scores ranging from 0 to 11, and scores ranging from 0 to 3, 4 to 7 and 8 to 11 indicating low, medium, and high quality, respectively. The detailed items of the AHRQ and the Newcastle–Ottawa Scale are shown in Supplementary Material 2.

### Data analysis

Stata 12.0 software was used to analyze all the data, and odds ratios (ORs) were calculated as the effect size meanwhile 95% confidence intervals (CIs) were provided. Heterogeneity was tested by *I*^2^, and *I*^*2*^ > 50% indicated high heterogeneity while *I*^*2*^ < 50% indicated low heterogeneity [34]. Pooled effect size was analyzed using a fixed-effects model if *I*^*2*^ < 50%. Subgroup and sensitivity analysis were used to analyze the sources of heterogeneity if *I*^*2*^ > 50%, and subsequently, a random effects model was used. To assess risk factors for FOF, the odds ratios (ORs) and 95% CIs, which reported the association between risk factors and FOF in eligible studies, were extracted to estimate the pooled effect size in meta-analyses using a fixed-effects model if *I*^*2*^ < 50%, or a random effects model if *I*^*2*^ > 50%. Publication bias was assessed through funnel plots, Egger’s test and Begg’s test [35].

## Results

### Study process

**Fig. 1:**
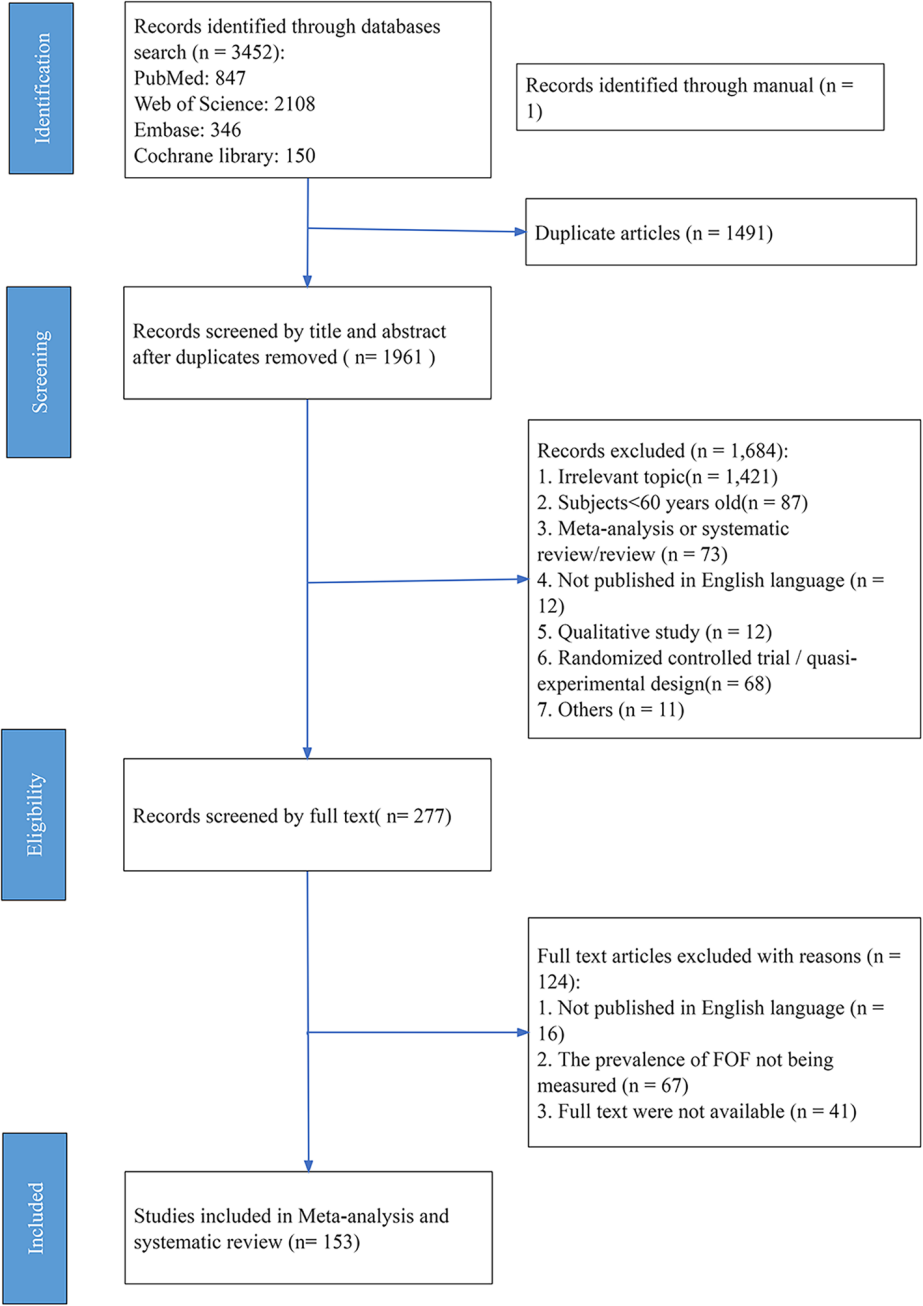
PRISMA flow diagram of literature search and selection of included studies for meta-analysis

A total of 3452 studies were retrieved from databases and manual searches, and 1491 duplicate studies were eliminated. A total of 277 studies that met the inclusion criteria were selected after screening titles and abstracts, 124 of which were excluded after screening the full text: 16 did not publish in English, 67 did not report the prevalence of FOF, and 41 did not provide the full text. Finally, 153 studies with 200,033 participants from 38 countries were included and analyzed (shown in Fig. 1).

### Characteristics of the included studies

The characteristics of the 153 studies, 64(41.83%) of which were of medium quality and 89(58.17%) of which were of high quality, are summarized in Table 1. The publication years of all studies ranged from 1994 to 2023, and there were 200,033, with 112,697(56.34%)female. The details of the 153 studies are shown in Supplementary Material 3.

**Table 1:** Characteristics of included studies (*N* = 153)

First author/ publication years	Countries	Type of studies	Instruments	Subjects	Age [mean ± SD / min–max, years]	Female (%)	Sample size(n)	FOF (%)	Quality of studies
Goh 2016 [36]	Malaysia	Cohort study	FES-I	Patients	≥ 60 years old; stroke: 66.6 ± 6.9; no-stroke:67.3 ± 4.5	42.40%	125	72.80%	6^b^
Damar 2021 [37]	Turkey	Cross-sectional study	FES	Patients	≥ 60 years old; 66.68 ± 5.57	61.60%	211	68.72%	6
Zhang 2021 [12]	China	Cross-sectional study	Are you afraid of falling? (yes/no)	Patients	≥ 65 years old; 75.2 ± 6.4	52.98%	285	56.49%	7
Lavedán 2018 [23]	Spain	Longitudinal study	Are you afraid of falling? (yes/no)	Community residents	≥ 75 years old; 81.3 ± 5.0	60.30%	640	37.03%	8^b^
Chen 2021 [38]	USA	Cohort study	In the last month/year, did you worry about falling down? (yes/no)	Community residents	≥ 65 years old	58.30%	5559	32.92%	8^b^
Turhan Damar 2018 [39]	Turkey	Cross-sectional study	FES	Patients	≥ 65 years old; 72.04 ± 7.76	69.60%	204	61.27%	9
Scarlett 2019 [40]	Britain	Cross-sectional study	FES-I	Community residents	≥ 65 years old; 76.0 ± 6.8	75%	117	72.65%	6
Tsonga 2016 [41]	Greece	Longitudinal study	Have you been worried or afraid that you might fall? (yes/no)	Patients	73 ± 5.28	83.82%	68	82.35%	4
James 2017 [42]	USA	Cross-sectional study	How afraid are you of falling? (“not at all afraid” = no FOF; “ somewhat afraid”, “fairly afraid” and “very afraid” = FOF)	Community residents	≥ 72 years old; 78.7 ± 5.0	60%	1169	58.94%	6
Rivasi 2020 [43]	Ireland	Longitudinal study	MFES	Community residents	≥ 60 years old; 72.7 ± 7.2	69.30%	563	15.10%	8^b^
Esbrí-Víctor 2017 [28]	Spain	Cross-sectional study	1. Are you afraid of falling?^a^ 2. Do you limit any household activities because you are frightened you may fall? 3. Do you limit any outside activities because you are frightened you may fall? 4. FES-I	Patients	≥ 70 years old; 78.4 ± 5.6	80.30%	183	76.50%	5
Simsek 2020 [24]	Turkey	Cross-sectional study	Do you have a fear of falling? (no FOF: “no fear”; FOF: “some fear” or “great fear”)	Community residents	≥ 80 years old; 84.1 ± 3.7	60.80%	1016	86.71%	7
Lach 2005 [44]	Britain	Longitudinal study	At the present time are you very fearful, somewhat fearful, or not fearful that you might fall (fall again)?	Community residents	≥ 60 years old; 75 ± 6.2	66%	890	28.20%	8^b^
Canever 2021 [45, 46]	Brazil	Cross-sectional study	FES-I	Community residents	≥ 60 years old	57.80%	308	48.38%	5
Murphy 2003 [47]	USA	Cohort study	Whether they ere afraid of falling (yes/no)	Community residents	79.3 ± 5.0 (ranged: 72–98)	100%	313	26.84%	9^b^
Oh 2017 [48]	Korea	Cross-sectional study	Do you usually have a feeling of FOF?(no FOF: “not at all”; FOF: “some” and “a lot”)	Community residents	73.51 ± 6.04 (ranged: 65–98)	60.90%	7924	75.63%	8
Birhanie 2021 [49]	Ethiopia	Cross-sectional study	FES-I	Community residents	≥ 60 years old; 67.9 ± 7.6	52.20%	481	59.88%	8
Oh-Park 2011 [50]	USA	Cohort study	1. Did you have fear of falling in the last 2 months or since the last interview?(yes/no)^a^; 2. MFES	Community residents	Aged ≥ 70; FOF:80.5 ± 5.2; no FOF:79.4 ± 5.3	53.16%	380	50.00%	9^b^
Rochat 2010 [51]	Switzerland	Cohort study	1. Are you afraid of falling (yes/no)^a^; 2. FES-I	Community residents	68.0 ± 1.4 (ranged: 65–70)	54.90%	860	29.65%	6^b^
Curcio 2009 [52]	Colombia	Cross-sectional study	Are you afraid of falling? (“no fear of falling”, “fear of falling alone”, and “activity restriction related to fear of falling”)	Community residents	≥ 60 years old; 70.90 ± 7.4	54.56%	1668	83.33%	7
Chang 2016 [53]	China-Taiwan	Cohort study	Are you presently afraid of falling?	Community residents	≥ 65 years old; 73.9 ± 5.8	43.57%	3824	53.40%	7^b^
Perez-Jara 2012 [22]	Spain	Cross-sectional study	Are you afraid of falling? (yes/no)	Patients	≥ 65 years old	NR	218	51.38%	7
Kumar 2014 [54]	Britain	Cross-sectional study	SFES-I	Community residents	≥ 65 years old; 72.9 ± 6.0	62.87%	1088	19.21%	9
Singh 2020 [55]	USA	Cross-sectional study	In the last month, did you worry about falling” (yes/no)	Community residents	≥ 65 years old	56.53%	4981	21.14%	9
Noh 2019 [56]	Korea	Cohort study	Are you afraid of falling? (“very fearful” “somewhat fearful” and “not fearful”)	Community residents	≥ 60 years old	58.27%	4280	64.37%	7^b^
Kim 2013 [57]	Korea	Cross-sectional study	Do you have a fear of falling(no fear, some, dread)	Community residents	≥ 65 years old; mean: 73.0	61.78%	9033	76.59%	7
Palagyi 2017 [58]	Australia	Cohort study	SFES-I	Community residents	≥ 65 years old; 75.8 ± 5.3	55.28%	322	32.92%	8^b^
Chang 2017 [59, 60]	China-Taiwan	Cohort study	Are you presently afraid of falling?”	Community residents	≥ 65 years old; 73.8 ± 5.8	43.58%	3814	53.41%	7^b^
Boyd 2009 [61]	USA	Cross-sectional study	How would you rate your fear of falling? (3–5 scores, 1–2 scores = ‘not or slightly afraid’, 3–5 = ‘moderately or very afraid’)	Community residents	≥ 65 years old	56.58%	1709	36.22%	7
Borges 2015 [62]	Brazil	Cross-sectional study	1. Are you presently afraid of falling?” (yes/no)^a^; 2. FES-I	Patients	≥ 60 years old	73.08%	104	54.81%	7
Visschedijk 2013 [13]	Netherlands	Cross-sectional study	1. Are you afraid of falling? (four answer options: “not at all”, “a little”, “quite a bit”, and “very much”^a^; 2. FES-I	Patients with hip fracture	≥ 65 years old; mean: 83.1	75.00%	100	27.00%	6
Perez-Jara 2009 [63]	Spain	Cross-sectional study	Are you fearful or worried about falling?(yes/no)	Patients	78.83 ± 6.48 (ranged: 60–92)	52.00%	200	50.00%	9
Visschedijk 2015 [64]	Netherlands	Longitudinal study	Are you afraid of falling? (“not at all”, “a little”, “quite a bit” and “very much);	Patients	Older patients, median (IQR): 82.4 (75.8–87.4)	70.71%	280	62.50%	7^b^
Vellas 1997 [65]	USA	Cohort study	Are you worried about falling again?(yes/no)	Patients	> 60 years old	65.30%	219	31.96%	8^b^
Park 2014 [66]	Korea	Cross-sectional study	FES-I (Korean version)	Community residents	> 60 years old; 69.4 ± 5.8	58.89%	883	38.84%	7
Austin 2007 [67]	Australia	Longitudinal study	1. Are you afraid of falling?(yes/ no)^a^; 2. FES	Female community residents	Mean:75.2 (ranged:70–85)	100%	1282	45.79%	8^b^
Auais 2016 [68]	Canada, Albania, Colombia, Brazil	Cross-sectional study	FES-I	Community residents	69.1 ± 2.8 (ranged:65–74)	51.70%	1875	53.71%	9
Mann 2006 [69]	Britain	Cross-sectional study	How worried they had been about having a fall in the last 4 weeks? (6-point Likert scale, ranging from none of the time to all of the time)	Female community residents	≥ 70 years old; 77.5 ± 4.76	100%	1691	59.02%	9
Canever 2022 [70]	Brazil	Cross-sectional study	FES-I (Brazil version)	Community residents	≥ 60 years old; 69.67 ± 6.99	57.79%	308	45.45%	7
Payette 2017 [71]	Canada	Cross-sectional study	1. FES-I^a^; 2. Are you afraid of falling, at least occasionally?	Community residents	≥ 65 years old; 75.72 ± 7.28	88.00%	25	80.00%	9
Jellesmark 2012 [72]	Denmark	Cross-sectional study	FES-I	Patients with hip fracture	Mean:81 (ranged 65–92)	78.79%	33	57.58%	8
Uemura 2012 [73]	Japan	Cross-sectional study	Are you afraid of falling? (“a little” or “not at all” = no FOF, “very much” or “somewhat” = FOF)	Community residents	≥ 65 years old; FOF:76.2–86.8; no FOF: 73.786.7	51.49%	101	53.47%	9
Howland 1998 [74]	USA	Cross-sectional study	1. How afraid are you that you will fall and hurt yourself in the next year? 2. Are there things you don’t do because you might fall? 3. Are there things you have stopped doing because you are worried that you might fall?	Community residents	76.3 ± 7.9 (ranged: 62–93)	77.00%	266	54.89%	8
Clemson 2015 [75]	Australia	Longitudinal study	Whether they were afraid of falling? (not at all afraid, somewhat afraid, fairly afraid, or very afraid)	Community residents	Mean:73.4 ( ranged 65–94)	53.30%	855	14.50%	8
Zijlstra 2007 [76]	Netherlands	Cross-sectional study	Are you afraid of falling?(never, almost never, sometimes, often or very, often)	Community residents	≥ 70 years old; 77.1 ± 4.9	59.94%	4031	54.23%	9
Jefferis 2014 [77]	Britain	Cross-sectional study	At the present time are you afraid that you may fall over?” (very fearful and somewhat fearful were compared to not fearful)	Male community residents	78.3 ± 4.6 (ranged: 71–92)	0%	1593	15.94%	8
Franzoni 1994 [78]	Italy	Longitudinal study	Are you afraid of falling? (FOF: “very fearful” and “somewhat fearful”; no -FOF: “not fearful)	Patients	83.1 ± 7.6	77.78%	54	46.30%	7^b^
Bruce 2002 [79]	Australia	Cross-sectional study	Are you afraid of falling? (yes/no)	Female community residents	≥ 70 years old; 75.2 ± 2.7	100%	1500	34.00%	9
Rossat 2009 [80]	France	Cross-sectional study	Are you afraid of falling?	Community residents	≥ 65 years old; 74.7 ± 4.4	57.50%	1189	34.06%	7
Kressig 2001 [81]	USA	Cross-sectional study	1. FES^a^; 2. ABC	Community residents	80.9 ± 6.2 (range: 70–98)	94.08%	287	47.74%	9
Kornfield 2017 [82]	USA	Longitudinal study	FFQ	Patients with hip fracture	78.8 ± 8.7 (ranged:60–101)	77.20%	456	45.18%	7^b^
Viljanen 2012 [83]	Finland	Longitudinal study	Are you afraid of falling? (“never” = no FOF; “occasionally”, “often” or “constantly” = FOF)	Female community residents	Ranged: 63–76	100%	434	39.86%	7^b^
Frankenthal 2021 [84]	Israel	Cross-sectional study	Are you afraid of falling? (yes/no)	Community residents	≥ 65 years old	57.26%	3159	44.82%	9
Park 2017 [85]	Korea	Cross-sectional study	Are you afraid of falling? (“very much”, “some-what”, and “not at all”)	Community residents	≥ 65 years old	56.87%	10,527	75.71%	8
Mane 2014 [86]	India	Cross-sectional study	SFES-I	Community residents	> 60 years old (ranged: 60–96)	48.00%	250	33.20%	6
Goldberg 2022 [87]	USA	Cross-sectional study	Do you have a fear of falling?(yes/no)	Community residents	≥ 60 years old; FOF:72.3 ± 9.6; no FOF: 71.8 ± 7.4	78.41%	88	27.27%	6
Wang 2022 [88]	China	Cross-sectional study	SFES-I (Chinese version)	Community residents	≥ 60 years old; 70.91 ± 6.71	68.80%	669	88.49%	9
Zheng 2016 [89]	China	Cross-sectional study	MFES	Patients	≥ 60 years old; 69.6 ± 8.3	82.05%	117	85.47%	9
Bertera 2008 [90]	USA	Cross-sectional study	Did you fear falling in the past year?( “everyday”, “once/twice per week”, “once/twice per month” and “a few times” = FOF, “never” = no FOF)	Community residents	≥ 65 years old	NR	3474	39.00%	6
Thiamwong 2017 [91]	Thailand	Cross-sectional study	1. Are you afraid/fear of falling?^a^; 2. How afraid/fear you are that you will fall?	Community residents	≥ 60 years old; 71.11 ± 7.73	64.51%	386	36.01%	7
Viljanen 2013 [92]	Finland	Longitudinal study	Are you afraid of falling? (“never” = no FOF; “occasionally”, “Often” or “constantly” = FOF)	Female community residents	Ranged: 63–76	100%	434	39.86%	7
Choi 2015 [20]	Korea	Cross-sectional study	Are you afraid of falling?(“not at all”, “a little”, and “very much”)	Community residents	≥ 65 years	58.72%	4247	77.51%	7
Taghadosi 2018 [93]	Iran	Cross-sectional study	FES-I	Community residents	> 60 years old; 68.76 ± 6.24	74.40%	414	90.34%	9
Vitorino 2019 [94]	Brazil, Portugal	Cross-sectional study	FES-I	Community residents	> 61 years old; 72.00 ± 7.69	74.41%	340	72.35%	9
Topuz 2014 [95]	Turkey	Cross-sectional study	In general, are you afraid of falling? ( not at all, a little, quite a bit, very much)	Community residents	> 61 years old; community dwelling: 70.07 ± 5.75; retirement village:82.37 ± 8.12	24.40%	86	30.23%	6
Sharaf 2008 [96]	Egypt	Cross-sectional study	1. Are you afraid of falling? (1 score “not at all afraid” to 4 score “severely afraid”^a^; 2. MFES	Community residents	73.21 ± 8.86 (ranged:60–102)	60.10%	208	52.88%	7
Chang 2010 [97]	China-Taiwan	Cross-sectional study	Are you afraid of falling? (yes/no)	Community residents	73.6 ± 5.9 (ranged:65–102)	43.96%	4056	53.43%	9
Ivanovic 2018 [98]	Serbia	Cross-sectional study	FES-I	Community residents	75.04 ± 5.85 (ranged:65–94)	59.00%	400	55.75%	7
Kulkarni 2020 [99]	India	Cross-sectional study	1. Are you afraid of falling?; 2. FES-I^a^	Community residents	≥ 60 years old; 68.00 ± 7 .0	60.50%	344	40.99%	9
Lee 2020 [7]	Korea	Longitudinal study	Are you usually afraid of falling? (yes/no)	Community residents	≥ 65 years old; 72.96 ± 6.28	58.10%	4104	70.54%	10
Sitdhiraksa 2021 [100]	Thailand	Cross-sectional study	FFQ	Community residents	≥ 60 years old; 70.3 1 ± 6.55	74.29%	210	25.24%	7
Lee 2021 [5]	Korea	Longitudinal study	Whether they had fear of falling?(‘no fear of falling’, ‘fear of falling but no related activity restriction’, and ‘fear-related activity restriction’ groups)	Community residents	≥ 60 years old	49.47%	2933	50.63%	9
Deshpande 2008 [101]	Italy	Cross-sectional study	SAFE	Community residents	≥ 65 years old; 75.9 ± 6.4	55.42%	848	6.96%	9
Savas 2019 [10]	Turkey	Cross-sectional study	Whether they were afraid of falling	Patients	≥ 65 years old; 76.7 ± 7.6	47.03%	555	22.16%	9
Brodowski 2022 [102]	Germany	Cross-sectional study	the Survey of Activities and Fear of Falling in the Elderly questionnaire 0 (“not worried”) and 3 (“very worried”)	Patients	80.09 ± 6 ( ranged:65–92)	68.37%	98	54.08%	8
Malini 2016 [103]	Brazil	Cross-sectional study	FES-I (Brazilian version)	Community residents	≥ 65 years old; 76.7 ± 7.03	70.22%	742	52.02%	7
Tomita 2018 [104]	Japan	Cross-sectional study	Are you afraid of falling?	Community residents	Ranged: 60–92; man: 70.1 ± 6.4; women: 69.8 ± 6.1	58.53%	844	36.49%	7
Lim 2011 [105]	Korea	Cross-sectional study	What extent are you afraid of falling?(“not at all” = no FOF; “slightly”, “somewhat” and “very much” = FOF)	Community residents	73.5 ± 6.3 (ranged:65–96)	59.66%	828	67.39%	9
Doi 2012 [106]	Japan	Cross-sectional study	Whether they had a fear of falling?(yes/no)	Female community residents	80.7 ± 5.4 (ranged:65–95)	100%	262	62.21%	6
Murphy 2002 [107]	USA	Cross-sectional study	Whether they had a fear of falling?(yes/no)	Community residents	79.6 ± 5.3(ranged:72–98)	73%	1064	42.86%	8
Chu 2011 [27]	China-Taiwan	Cross-sectional study	Are you afraid of falling? (“never” and “almost never” = no FOF, “sometimes”, “often” and “very often” = FOF)	Community residents	82.1 ± 5.1 (ranged:67–99)	NR	371	25.34%	9
Nawai 2022 [108]	Thailand	Cross-sectional study	SFES-I	Community residents	≥ 60 years old; 70.9 ± 6.9	71.23%	365	56.16%	7
Drummond 2022 [109]	Brazil	Cross-sectional study	FES-I	Community residents	> 65 years old	68.38%	291	51.89%	9
Kurkova 2020 [110]	Czech Republic	Cross-sectional study	FES-I	Community residents	≥ 65 years old	75.36%	349	79.66%	7
Gupta 2022 [14]	India	Cross-sectional study	FES-I	Patients with diabetes	68.26 ± 5.9 (ranged:60–80)	30.70%	316	39.24%	7
Boltz 2014 [111]	USA	Cross-sectional study	How fearful they were of falling? (0 to 4 score, 0–1 score = no FOF, ≥ 2 score = FOF)	Patients	81.7 ± 7.7 (ranged:70–97)	58.54%	41	68.29%	7
Brustio 2018 [112]	Italy	Cross-sectional study	FES-I	Community residents	70.87 ± 5.16 (ranged: 60 -80)	67.11%	76	68.42%	7
Hewston 2018 [113]	Jamaica	Cross-sectional study	FES-I	Patients with diabetes	Ranged: 65–74	44.55%	761	84.23%	6
Kakhki 2018 [114]	Iran	Cross-sectional study	FES-I (Persian version)	Patients	68.62 ± 6.82 (ranged:60–90)	54.82%	301	31.56%	6
Makino 2021 [115]	Japan	Cohort study	Are you afraid of falling? ( FOF: “very much” or “some what”; no FOF: “a little” or “not at all	Community residents	≥ 65 years old; 71.1 ± 4.7	51.64%	2469	41.47%	8^b^
Akosile 2021 [116]	Nigeria	Cross-sectional study	MFES	Community residents	≥ 65 years old; group assisted living facility: 78.98 ± 9.36; group community dwelling: 71.28 ± 7.85	65.79%	114	55.26%	5
Nguyen 2020 [11]	Vietnam	Cross-sectional study	Are you afraid of falling? (yes/no)	Patients	≥ 60 years old	60.00%	405	88.15%	9
Vitorino 2017 [117]	Brazil	Cross-sectional study	FES-I (Brazilian version)	Community residents	≥ 60 years old	67.65%	170	66.47%	8
Teixeira 2019 [118]	Portugal	Cross-sectional study	Do you have fear of falling? (yes/no)	Community residents	101.0 ± 1.5, (ranged:100–107)	86.24%	109	78.90%	9
Liu 2021 [17]	USA	Longitudinal study	In the last month, did you worry about falling down? (yes/no)	Community residents	≥ 65 years old	42.94%	864	9.26%	8^b^
Gottschalk 2020 [119]	Germany	Cross-sectional study	SFES-I(German version)	Patients	≥ 70 years old; 78.67 ± 5.31	73.46%	309	66.02%	6
Umegaki 2021 [19]	Japan	Cross-sectional study	FES-I (Japanese version)	Community residents	72.4 ± 4.6 (ranged:65–85)	46.29%	458	86.46%	6
Vo 2020 [30]	Vietnam	Cross-sectional study	FES-I	Community residents	≥ 60 years old	58.34%	725	62.48%	9
Shahid 2020 [120]	Pakistan	Cross-sectional study	FES-I	Community residents	> 65 years old; 70.03 ± 4.52	19.64%	336	75.89%	8
Liu 2015 [121]	China- Hong Kong	Cross-sectional study	FES-I (Chinese version)	Community residents	≥ 65 years old	75. 28%	445	64.72%	9
Sakurai 2021 [122]	Japan	Cohort study	Are you afraid of falling during everyday activities?(yes/no)	Community residents	Older adult; 73.3 ± 5.4	75.00%	184	53.80%	8^b^
Asai 2017 [18]	Japan	Cross-sectional study	Are you afraid of falling? (yes/no)	Community residents	71.9 ± 3.9 (ranged:65–80)	56.00%	260	22.31%	8
Du 2022 [123]	China	Cross-sectional study	ABC (Chinese version)	Female community residents	64.9 ± 2.8 (ranged: 60 -70)	100%	1101	18.80%	8
Park 2022 [124]	Korea	Longitudinal study	Are you usually afraid of falling”(“not at all” and “a little” = no FOF, “very much” = FOF)	Community residents	≥ 65 years old; 71.47 ± 5.28	49.98%	2691	16.69%	8^b^
Arfken 1994 [125]	USA	Cohort study	At the present time, are you very fearful, somewhat fearful or not fearful that you may fall (again)?”	Community residents	≥ 65 years old	67.08%	890	28.88%	7^b^
Chou 2007 [126]	China	Cohort study	Whether they had limited their outdoor activities because of a fear-of-falling?(‘0’ = “no limitation of activity” and ‘1’ = “reported limitation”)	Patients	≥ 65 years old; 72.6 ± 5.5	52.02%	321	18.07%	8^b^
Bahat Öztürk 2021 [25]	Turkey	Cross-sectional study	Are you afraid of falling?(yes/no)	Community residents	≥ 60 years old; 74.9 ± 6.9	67.90%	1021	44.56%	7
Arani 2020 [127]	Iran	Cross-sectional study	FES-I	Patients with diabetes	≥ 60 years old	40.30%	134	51.49%	7
Yoshikawa 2019 [16]	USA	Cross-sectional study	How fearful are you of falling: (no FOF: “not at all”; FOF: “a little”, “somewhat”, “a lot”)	Community residents	≥ 60 years old; 76.45 ± 7.79	82.38%	522	83.33%	7
Ren 2022 [128]	China	Cross-sectional study	ABC (Chinese version)	Female community residents	64.9 ± 2.8 (ranged: 60 ~ 70)	100%	1114	19.03%	6
Katsumata 2011 [129]	Japan	Cross-sectional study	Have you restricted your activities because you have been anxious of falling in the past year?(yes/no)	Community residents	≥ 65 years old	52.93%	648	29.32%	8
Friedman 2002 [130]	USA	Cross-sectional study	1. Apart from being in a high place, in the past 12 months, have you been worried or afraid that you might fall?; 2. Do you ever limit your activities, for example, what you do or where you go, because you are afraid of falling?	Community residents	Mean:72.6 (rang: 65.9–86.3)	58.60%	2212	20.75%	8
Löppönen 2022 [131]	Finland	Cross-sectional study	Are you afraid of falling? (no FOF: “never”; FOF: “occasionally”, “often” and “constantly”)	Community residents	≥ 75 years old	59.92%	479	71.82%	5
Lach 2020 [132]	USA	Cross-sectional study	1. if they were somewhat, very or not at all concerned about having a fall?^a^; 2. NHFSS	Community residents	86.2 ± 7.4	74.20%	225	50.67%	7
Van Haastregt 2008 [133]	Netherlands	Cross-sectional study	Are you afraid of falling? ( “never” and “almost never” = no FOF, “sometimes” = mild FOF, “often”and “very often” = severe FOF)	Community residents	77.6 ± 4.8 (ranged:70–92)	71.85%	540	44.81%	7
Schroeder 2022 [134]	Germany	Cohort study	1. Do you actually have a FOF? ( yes/no)^a^; 2. FES-I	Female community residents	≥ 60 years old; 72.5 ± 7.1	100%	431	56.84%	6^b^
Sakurai 2017 [135]	Japan	Cohort study	Are you afraid of falling? (yes/no)	Community residents	≥ 60 years old; 74.0 ± 5.3	77.78%	117	50.43%	8^b^
Aburub 2020 [136]	Canada, Albania, Colombia, Brazil	Cohort study	FES-I	Patients	Ranged: 65–74; group cancer: 69.3 ± 2.9; group healther:69.2 ± 2.7	51.99%	352	23.30%	7
Gagnon 2005 [137]	Canada	Cross-sectional study	1. Are you afraid of falling? (not at all afraid, slightly afraid, moderately afraid, or very afraid)^a^; 2. MFES	Patients	78.2 ± 8.9 (ranged:60–97)	86.67%	105	45.71%	9
Visschedijk 2014 [138]	Dutch	Cross-sectional study	FES-I	Patients with hip fracture	≥ 65 years old; 83.1 ± 8.3	75.00%	100	50.00%	8
Jaatinen 2022 [139]	Finland	Cohort study	Do you have a fear of falling? or “Are you afraid of falling?(yes/no)	Patients with hip fracture	≥ 65 years old	71.94%	916	49.34%	7^b^
Martínez-Arnau 2021 [140]	Spain	Cross-sectional study	FES-I	Community residents	≥ 70 years old; 77.8 ± 4.9	70.31%	229	51.09%	7
De Roza 2022 [9]	Singapore	Cross-sectional study	SFES-I	Community residents	≥ 65 years old; mean:78.3	59.70%	360	60.83%	7
Pohl 2015 [141]	Sweden	Cross-sectional study	1. Are you afraid of falling? (rarely/never, sometimes, or often/always)^a^; 2. ABC; 3. SAFFE	Community residents	79.5 ± 3.7 (ranged: 75–93)	72.17%	230	46.09%	6
Trevisan 2020 [142]	Italy	Cohort study	Whether they were afraid of falling? (yes/no)	Community residents	≥ 65 years old; 75.4 ± 7.3	58.93%	2625	46.10%	8^b^
Choi 2017 [143]	Korea	Cohort study	Are you afraid of falling? (“not at all” and “slightly” = no FOF, very much = FOF)	Female community residents	≥ 70 years old; 77.17 ± 5.64	100%	1560	38.46%	8^b^
Merchant 2020 [144]	Singapore	Cross-sectional study	Are you afraid of falling? (yes/no) or( “yes, a lot”)	Community residents	≥ 60 years old; 73 ± 8	79.31%	493	69.17%	7
Asai 2022 [145]	Japan	Longitudinal study	Are you afraid of falling? (yes/no)	Community residents	≥ 65 years old	66.79%	530	53.96%	8^b^
Yang 2020 [146]	China	Cross-sectional study	MFES	Patients	78.9 ± 5.5 (ranged:70–93)	74.47%	47	53.19%	7
Freiberger 2022 [147]	Austria, Germany, Israel, Italy, Netherlands, Poland and Spain	Cohort study	Are you afraid of falling? (“not at all concerned”, “somewhat concerned”, “fairly concerned” and “very concerned”)	Patients	≥ 75 years old; 79.0 ± 6.0	63.50%	389	78.66%	7^b^
Peterson 1999 [148]	USA	Cross-sectional study	FES	Community residents	76.2 ± 7.9 (ranged:62–93)	78.00%	270	70.00%	9
Sawa 2023 [149]	Japan	Cohort study	Are you afraid of falling? (no FOF: “not at all”, “a little”; FOF: “somewhat”, “very much”)	Community residents	≥ 65 years old; 73.5 ± 5.5 year	51.86%	9372	48.90%	7^b^
You 2023 [150]	China	Cross-sectional study	At present are you afraid that you may fall over? (yes/no)	Community residents	≥ 60 years; 72.9 ± 8.4 years	50.14%	7,774	31.61%	7
Garbin 2023 [151, 152]	USA	Longitudinal study	In the last month, did you worry about falling down? (yes/no)	Community residents	≥ 65 years old; FOF:79.62 ± 7.80; no FOF:75.28 ± 7.25	51.91%	680	11.32%	8^b^
Liu 2023 [153]	USA	Longitudinal study	Did you worry about falling down in the last month?	Community residents	≥ 65 years old	58.17%	5950	28.64%	8^b^
Scheffers-Barnhoorn 2023 [154]	Netherlands	Cohort study	SFES-I	Patients with hip fracture	≥ 70 years old; 81.9 ± 7.1	68.69%	444	57.21%	7^b^
Prado 2023 [155]	Brazil	Cross-sectional study	FES-I	Community residents	≥ 60 years	57.79%	308	45.45%	7
Zhang 2023 [156]	China	Cross-sectional study	SFES-I	Community residents	≥ 65 years old	66.17%	541	55.27%	8
Chu 2023 [157]	China-Taiwan	Cross-sectional study	ABC	Patients	≥ 65 years old; 72.04 ± 5.53	71.56%	211	23.22%	7
Canever 2021 [45, 46]	Brazil	Cross-sectional study	FES-I	Community residents	≥ 60 years old	57.79%	308	45.45%	8
Wang 2022 [88]	China	Cross-sectional study	SFES-I	Community residents	≥ 60 years old; 70.91 ± 6.71	68.80%	669	88.49%	5
Freiberger 2022 [147]	Austria, Germany, Israel, Italy, Netherlands, Poland and Spain	Cohort study	Are you afraid of falling? (“not at all concerned”, “somewhat concerned”, “fairly concerned” and “very concerned”)	Patients	> 75 years old; 79.0 ± 6.0	63.50%	389	78.66%	7^b^
Siefkas 2022 [158]	USA	Longitudinal study	Whether participants had worried about falling down in the last month?(yes/no)	Community residents	≥ 65 years old	55.04%	2858	24.32%	7^b^
Korenhof 2023 [159]	Centers:United Kingdom, Greece, Croatia, Netherlands and Spain	Cross-sectional study	SFES-I	Community residents	≥ 70 years old; 79.7 ± 5.6	60.58%	2189	49.93%	6
Garbin 2023 [151, 152]	USA	Cohort study	In the last month, did you worry about falling down? (yes/no)	Community residents	≥ 65 years old; FOF:78.65 ± 7.75;no FOF:76.02 ± 7.21	57.25%	5151	37.64%	8^b^
Dos Santos 2023 [160]	Brazil	Cross-sectional study	FES-I (Brazilian version)	Community residents	≥ 60 years old; 70.11 ± 7.22	66.34%	410	43.90%	8
DiGuiseppi 2022 [161]	USA	Cohort study	SFES-I	Community residents	Ranged: 65–79	52.94%	2941	18.60%	7^b^
Badrasawi 2022 [162]	Palestine	Cross-sectional study	FES-I	Community residents	70.5 ± 5.7 (ranged:65–98)	68.50%	200	49.00%	8
McKay 2022 [163]	USA	Cross-sectional study	FES	Community residents	≥ 65 years old; 79.35 ± 5.65	66.94%	242	30.17%	6
Luo 2022 [164]	USA	Longitudinal study	In the last month, did you worry about falling down? (yes/no)	Community residents	≥ 65 years old; 78 ± 7.73	58.11%	6376	29.22%	8^b^
Shiratsuchi 2022 [165]	Japan	Cross-sectional study	Are you afraid of falling? (yes/no)	Community residents	73.3 ± 5.4 (ranged: 69–77)	59.90%	9759	35.41%	6
Turhan Damar 2022 [166]	Turkey	Cross-sectional study	FES-I (Turkish version)	Patients	74.77 ± 7.78 (ranged:65–100)	47.92%	409	88.75%	8
Dhar 2022 [167]	India	Cross-sectional study	FES-I (Hindi version)	Patients	> 60 years old	49.06%	795	42.01%	6

*FES-I* The Falls Efficacy Scale International, *FES* The Fall Efficacy Scale, *MFES* The Modified Falls Efficacy Scale, *SFES-I* The Short Falls Efficacy Scale-International, *ABC* Activities-Specific Balance Confidence Scale, *FFQ* The Fear of Falling Questionnaire, *SAFE* The Survey of Activities and Fear of Falling in the Elderly questionnaire, *NHFSS* The Nursing Home Falls Self-efficacy Scale, *SAFFE* Survey of Activities and Fear of Falling in the Elderly

^a^The prevalence of FOF assessed by the tool was analyzed in meta-analysis

^b^The Newcastle–Ottawa Scale score

### Global prevalence of FOF

The global prevalence of FOF among older adults widely ranged from 6.96% to 90.34% in the 153 studies. The overall prevalence of FOF was 47.80% [95% CI: 47.7%–48.0%], with high heterogeneity (χ^2^ = 50,648.15, I^2^ = 99.7%, *p* < 0.001). A random effects model was then constructed, and the results showed that the overall prevalence of FOF was 49.60% [95% CI:45.9%–53.2%, *I*^2^ = 99.7%, *p* < 0.001] (as shown in Supplementary Material 3).

### Subgroup analysis

The subgroup analysis by region, country, subject and instrument used is shown in Table 2. The estimates of the pooled prevalence of FOF were higher in Africa and Asia than in other regions, at 56.80% and 52.90%, respectively; were higher in developing countries (53.40%) than in developed countries (46.7%); and were higher in patients (52.20%) than in community residents (48.40%). Moreover, there is a difference in the prevalence of FOF among different instruments.

**Table 2:** Subgroups analyses by regions, countries, subjects and instruments

Subgroups	Number of included studies	FOF				
		Prevalence	95%CI		I 2	*P* value
**Regions**						
Multi center studies	6	59.40%	45.80%	73.00%	99.10%	< 0.001
Africa	3	56.80%	52.20%	61.50%	36.30%	< 0.001
Asia	64	52.90%	47.70%	58.20%	99.70%	< 0.001
Europe	36	49.30%	42.40%	56.20%	99.30%	< 0.001
America	40	44.10%	38.30%	49.80%	99.60%	< 0.001
Oceania	4	31.80%	17.40%	46.20%	99.00%	< 0.001
**Developed or developing countries**						
Multi center studies	6	59.40%	45.80%	73.00%	99.10%	< 0.001
Developing countries	53	53.40%	47.00%	59.80%	99.50%	< 0.001
Developed countries	94	46.70%	42.20%	51.30%	99.80%	< 0.001
**Research subjects**						
Patients	47	52.20%	45.50%	58.90%	99.10%	< 0.001
Community residents	106	48.40%	44.10%	52.70%	99.80%	< 0.001
**Instruments**						
Single question	88	0.473	0.428	0.519	99.80%	0.000
FES-I	36	0.576	0.511	0.641	98.70%	0.000
SFES-I	11	0.539	0.357	0.721	99.80%	0.000
FES	5	0.556	0.405	0.706	96.90%	0.000
MFES	4	0.522	0.121	0.923	99.30%	0.011
ABC	3	0.193	0.177	0.208	2.30%	0.000
Others	6	0.341	0.210	0.472	99.00%	0.000

*FOF* Fear of falling, *Single question* “Are you afraid of falling?”, “Are you fearful or worried about falling?”, or “Whether they had a fear of falling”, *FES-I* The Fall Efficacy Scale International scale, *SFES-I* The Shortened Version of the Falls Efficacy Scale International, *FES* The Fall Efficacy Scale, *MFES* The Modified Fall Efficacy Scale, *ABC* The Activities-Specific Balance Confidence, Others: *FFQ* The Fear of Falling Questionnaire, *SAFFE* The Survey of Activities and Fear of Falling in the Elderly questionnaire

### Risk factors for FOF

A total of thirty-eight risk factors for FOF were analyzed, and twenty-eight of them were significant significantly associated FOF (*p* < 0.05), including demographic characteristics (e.g., female sex, age (70–84 years), low education level, living alone, high BMI, etc.), physical function(e.g., using walking aid, frailty status, poor perceived health, Timed Up and Go test results (abnormal), balance problems), chronic diseases(e.g., diabetes, hearing impairment, visual impairment, body pain, dizziness, number of chronic diseases, etc.) and mental problems (e.g., anxiety and depression), while ten of them were not (*p* > 0.05), as shown in Table 3.

**Table 3:** Pooled risk factors of fear of falling(FOF)

**Risk factor**	**Number of included studies**	**Effect model**	**OR**	**95% CI**	***I***^**2**^	***P*-value**
**Demographic characteristics**						
Female	34	Random	2.361	2.053- 2.716	86.30%	< 0.001
Age (70–84 years)	17	Random	1.328	1.229–1.434	91.10%	< 0.001
Unmarried	3	Random	1.336	0.648–2.755	76.80%	0.432
Low education	11	Random	1.268	1.104–1.458	60.40%	0.001
Living alone	13	Random	1.228	1.026–1.469	73.40%	0.025
History of fall	19	Random	2.390	1.731–3.299	89.80%	< 0.001
Falling in last one year	16	Random	2.709	1.992–3.683	90.50%	< 0.001
High BMI	8	Random	1.073	1.026–1.122	82.80%	0.002
**Physical function**						
Using walking aid	11	Random	2.517	1.729–3.665	77.90%	< 0.001
Frailty	5	Random	4.262	1.714–10.600	89.10%	0.002
ADL (Dependent)	8	Random	1.778	1.258–2.513	80.80%	0.001
Limitation of IADL	8	Random	2.085	1.412–3.079	91.8%%	< 0.001
Limitation of BADL	3	Random	2.439	0.652–9.119	98.00%	0.185
Mental health Statue (SF-36)	2	Random	0.920	0.829–1.021	78.70%	0.116
Poor perceived health	13	Random	2.407	1.724–3.363	94.30%	< 0.001
Timed Up and Go test (abnormal)	8	Random	1.222	1.097–1.362	81.70%	< 0.001
Clinical gait abnormality	7	Random	1.415	0.858–2.333	74.00%	0.174
Problems with balance	7	Random	1.854	1.325–2.595	63.00%	< 0.001
Needs of mobility assistance	3	Random	1.963	1.082–3.560	74.00%	0.026
**Chronic diseases**						
Diabetes mellitus	6	Fixed	1.298	1.174–1.435	32.20%	< 0.001
Hypertension	4	Random	1.151	0.791–1.675	83.50%	0.464
Hearing impairment	3	Fixed	1.476	1.241–1.754	0.00%	< 0.001
Visual impairment	9	Random	1.695	1.280–2.244	74.20%	< 0.001
Body pain	4	Random	1.881	1.342–2.638	73.70%	< 0.001
Dizziness	4	Random	2.894	1.141–7.339	90.40%	0.025
Cardiopulmonary pattern	3	Random	1.510	0.943–2.418	68.20%	0.086
Musculoskeletal pattern	3	Fixed	1.766	1.382–2.256	8.20%	< 0.001
Cognitive impairment	6	Random	1.567	0.822–2.986	92.90%	0.172
Cognition statue MMSE	6	Random	1.045	0.974–1.121	61.50%	0.218
Chronic diseases 1–2	5	Random	1.193	1.032–1.380	59.30%	0.017
Chronic diseases ≥ 2	7	Random	1.361	1.122–1.652	89.10%	0.002
Chronic diseases ≥ 3	3	Random	1.908	1.252–2.906	88.70%	0.003
Comorbidities ≥ 2	3	Random	0.968	0.686–1.366	44.60%	0.853
Types of medications ≥ 4	3	Random	1.681	1.028–2.748	65.30%	0.038
**Psychological factors**						
Anxious	7	Random	1.691	1.290–2.217	92.40%	< 0.001
Depression (GDS)	22	Random	1.516	1.354–1.698	85.10%	< 0.001
Depressive symptoms	6	Random	1.934	1.395–2.680	94.60%	< 0.001
**Social support**						
Low social support	4	Random	1.186	0.926–1.519	68.50%	0.176

*BMI* Body mass index, *GDS* Geriatric depression scale, *ADL* Activity of daily living, *SF-36* Short form health survey, *IADL* Instrumental activity of daily living, *BADL* The Barthel Activities of Daily Living Scale, *MMSE* The Mini-Mental State Examination

Cardiopulmonary pattern: chronic bronchitis or asthma, cardiac diseases, and tuberculosis. Musculoskeletal pattern: arthritis or rheumatism, chronic back problems, and osteoporosis

### Publication bias and sensitivity analysis

**Fig. 2:**
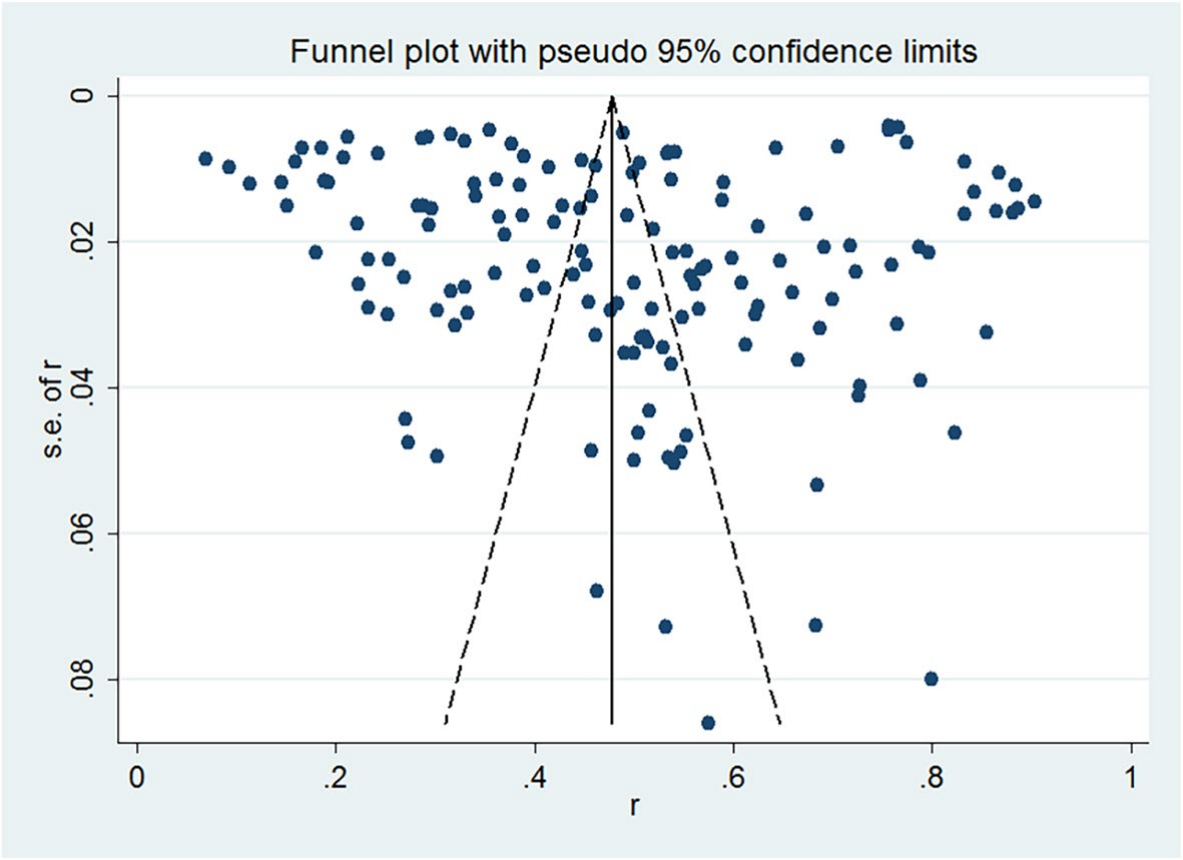
Funnel plot for assessing publication biases

**Fig. 3:**
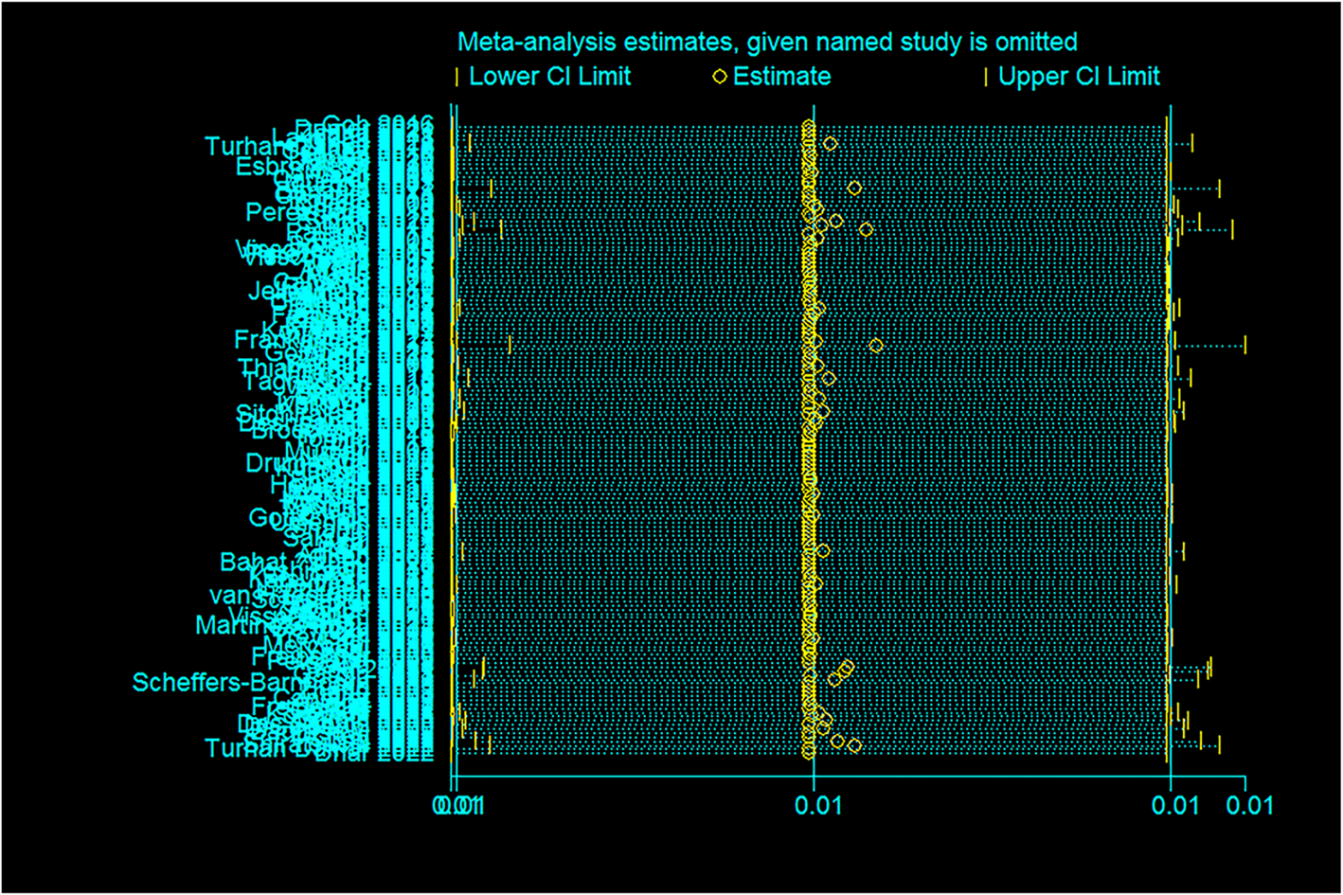
Sensitivity analysis of global prevalence of FOF

Begg’s test (z = 1, *p* = 0.320) and Egger’s test (t = 15.34, *p* < 0.001) revealed the potential publication bias of the included literature, and the funnel plot showed a asymmetry (shown in Fig. 2). However, the sensitivity analysis of this finding was robust (shown in Fig. 3).

## Discussion

This study was the first systematic review and meta-analysis to analyze the global prevalence of FOF among older adults, and to fully explore its potential risk factors. A total of 153 studies involving 200,033 participants from 38 countries revealed that the prevalence of FOF ranged widely from 6.96% to 90.34%, which was lower than that reported (ranging from 22.5% to 100%) among hip fracture patients [168], and the pooled global prevalence of FOF was high at 49.60%, which was similar to the results (44.6%) of previous research [25]. Subgroup analysis revealed that the pooled prevalence of FOF was higher in Africa and Asia than in other regions, higher in developing countries than in developed countries, and higher in patients than in community residents. In addition, twenty-eight potential risk factors were found to be significantly associated with FOF, mainly including demographic characteristics, physical function, chronic diseases and mental problems, which was the same as that reported in earlier studies [29, 169].

Overall, this study revealed that the global prevalence of FOF among older adults was high. One important reason was the increasing aging of the global population, which increased the prevalence of FOF among older adults. The WHO’s Aging and Health Report showed that in older adults, falls, as one of the common health conditions associated with aging, could lead to major public health problems and socioeconomic burdens [170], and FOF, as a fall-related mental problem, could increase older adults’ risk of falls, and these two factors could form a vicious cycle. Unlike in general older adults, the prevalence of FOF among patients after hip fracture tended to decrease within 4 weeks, approximately 12 weeks and over 12 weeks, at 50% to 100%, 47% to 59%, and 23% to 50%, respectively [168]. Moreover, the findings of this study revealed that the prevalence of FOF in Africa and Asia was high. Vo, et al. [169] reported that FOF among older adults Southeast Asia ranged from 21.6% to 88.2%, which was probably explained by a social environment that was unfriendly toward older adults, population aging, unbalanced economic conditions and a lack of familial support throughout urbanization. A previous study revealed that in developing countries, the high prevalence of FOF might be caused by low levels of education that prevent people from successfully managing FOF on their own, health caused by chronic diseases, inability to participate in social activities, and insufficient medical resources to properly manage both physical and psychological concerns [49], and inadequate service systems might have an impact on people’s ability to self-manage poor coping skills. Notably, although developed countries have better economic conditions, access to health care, educational opportunities, and social services than developing countries, some of them have high rates of FOF, such as the USA [16], Spain [28], Korea [20], and Japan [19]. It is likely that unhealthy lifestyles and diet habits lead to abnormal BMIs [54]. Ercan [171] reported that obesity could impact individuals’ posture and lead to balance problems, and obese females have higher FOF, higher activity restriction, and lower activity confidence than obese males. Earlier evidence demonstrated that older women with a high waist circumference had three times more likely to develop FOF than were those with a low waist circumference, which could alter the body’s center of gravity, further impair postural stability, and contribute to FOF [155]. However, another study showed that although BMI could slightly influence on body swing on unstable surfaces, obesity was not associated with FOF [171]. Furthermore, James [42] attempted to investigate the effect of the English language on fear of falling among Mexican-Americans in USA, but the results showed that not speaking or understanding English did not increase the incidence of FOF among those less than 80 years old, but it could affect activity restriction, to some extent.

Compared with community-dwelling residents, the prevalence of FOF was higher among those with chronic disease, especially patients with hip fracture [172], knee osteoarthritis [12], diabetes [173], etc. Previous studies have shown a high incidence of fear of falling in patients with hip fractures who underwent surgery involving knee replacement, total hip replacement or spinal surgery [37, 39], and FOF and cognitive impairment had a stronger impact on functional rehabilitation than did pain and depression [172]. In diabetic patients, symptoms of peripheral neuropathy, such as pain, feeling of ant walking, freezing, and burning, eventually impeded their ability to move and increased their likelihood of experiencing fear of falling [14]. Chronic pain has also been confirmed to m increase individuals’ susceptibility to FOF, and it plays a mediating role between FOF and poor physical performance [108]. Moreover, a qualitative study revealed that FOF gave patients with Parkinson’s disease (PD) a sense of insecurity, vulnerability and danger in daily activities, and when facing PD-related symptoms, such as rigidity, gait freezing or balance problems, positive emotions would help them successfully cope with FOF [174]. However, cardiopulmonary pattern (hypertension) and cognitive impairment were not significantly associated with FOF in this study, which was not consistent with previous studies [30], in which the relationship between cognitive impairment and FOF decreased due to the effect of high social support. In addition, the meta-analysis of risk factors in this study indicated that regardless of the number of chronic diseases, they negatively effected on FOF. On the one hand, multiple comorbidities can affect the multiorgan function of older adults, and on the other hand, due to a reduction in metabolic function, the side effects of treating this disease with multiple drugs can negatively impact on health.Therefore, multidisciplinary cooperation, including rehabilitation, pharmacy, nutrition, psychology, etc., can help to prevent and reduce FOF among older adults.

Demographic characteristics (etc., age, female sex, low education level, living alone, history of falls) are the well-known, significant factors of FOF among older adults. Birhanie [49] reported that compared with individuals aged 60 to 70 years, those aged more than 70 years were four times more likely develop FOF, which was similar to our results. Previous studies have shown that females had a greater risk of fear of falling than men do, and a decrease in estrogen among older women could cause osteoporosis, bone hyperplasia and a decrease in limb muscle mass, further leading to a weakened musculoskeletal system [49]. Additionally, those with back pain, mental health conditions and neurological disorders are more likely to develop chronic disease [175]. Thiamwong [91] reported that the female sex and low education were closely associated with fear of falling, and the latter was important for preventing individuals from engaging in FOF education and learning how to prevent it. Moreover, according to a previous investigation, nearly 80% of older adults with a history of falling had a high fear of falling, especially among those who were over 85 years old, for whom nearly 95% of the participants were adults [7]. Frankenthal [84] indicated that the prevalence of FOF (69.8%) among people with a history of falls was higher than that among people without a history of falls (41.4%). Notably, living alone was also a significant factor for FOF, while being unmarried not. Older adults who lived alone had no assistance in daily activities and had no else help in dangerous situations. However, interestingly, De Roza [9] reported that older adults who were married status had greater FoF than those who were never married, which could be explained by the fact that those who never married might have developed great independence at an early age. In addition, because of the great independence, older adults who unmarried might have greater psychological resilience and better ability to cope well with FOF. Furthermore, we also found that the low social support was not significantly related to FOF in this study. Dierking [176] noted that social support had both positive and negative effects on people’s health, and that familial conflict could increase the risk of FOF, but friend support had a positive effect on preventing FOF. Hence, we suggest that actively addressing family conflicts and more social networks should be considered in FOF prevention programs.

Physical function, such as using walking aid, frailty, dependent daily activities, and balance problems, was significantly related to FOF, which was consistent with the findings of Gadhvi [168]. Birhanie [49] showed that older adults who used walking aids were fourteen timed more likely to developing FOF than those who did not use them. De Roza [9] noted that older adults who used quad sticks had greater FOF than did those who used umbrellas or walking sticks and that the use of walking aids was closely related to frailty, which subsequently impacted FOF. Furthermore, previous studies had reported that FOF and its related activity restriction were associated with impaired gait, balance problems, frailty, sarcopenia, depressive symptoms, and mortality [5, 144, 177]. Moreover, consistent with a previous study [49, 147], depression and anxiety were found to be the most common, significant psychological risk factors for FOF in this study. A meta-analysis by Gambaro et al. [6], revealed that FOF might play a mediating role between depression and falls. In addition, social culture and attitudes regarding aging-related changes were found to be strongly associated with FOF [178]. For example, one of the important reasons for the high prevalence of FOF among Korean older adults was the use of public transportation, such as buses or subway [48]. Therefore, we suggest that in addition to improving physical function, increasing balance confidence and changing the incorrect cognition of FOF, social infrastructure (e.g., walking paths, public transportation), home environments(e.g., using automated LED lighting), and social service policies to prevent and reduce FOF in older adults should be considered to increase the prevalence of FOF and create an age-friendly society.

This study also had several limitations. First, due to the involved observational studies, there might be some compounding factors, which might bias to the results. Notably, a large sample of 153 studies with 200,033 subjects from 38 countries could be advantageous for guaranteeing the consistency and universal applicability of the results. Second, high heterogeneity in this work was found, caused in part by the subjects from various nations, living conditions, cultures and lifestyles. Finally, only three studies from Africa were analyzed, probably because the work included only English studies, which may have left out some important evidence in other languages. Hence, more studies should pay more attention to FOF among older adults who speak different languages in the future.

## Conclusion

This study as the first systematic review and meta-analysis provided substantial evidence that the global prevalence of FOF was high, and it was higher in developing countries than in developed countries, and higher in patients than in community residents. Twenty-eight potential risk factors, including demographic characteristics, physical function, chronic diseases and mental problems, were found a significant association with FOF. Policy-makers, health care providers and government officials should comprehensively evaluate the risk factors for FOF among older adults and formulate precise intervention measures to improve FOF based on the characteristics of different individuals. Firstly, multidisciplinary cooperation models should be established, including rehabilitation, psychology, pharmacology, etc, to help older patients normatively treat chronic diseases, strengthen drug safety management, and prevent drug abuse to reduce FOF. Secondly, a friendly living environment including improving exercise facilities and equipment and providing social support should be built to help older adults actively participate in social engagement. Finally, policy-makers should formulate the age-appropriate transformation system and intelligent health care system, optimize the health service model of older adults, actively develop the silver economy, and provide policy support and economic guarantee for promoting the physical and mental health of older adults.

### Supplementary Information

**Supplementary Material 1.**

**Supplementary Material 2.**

**Supplementary Material 3.**

**Supplementary Material 4.**

## Data Availability

The datasets used and/or analyzed during the current study are available from the corresponding author on reasonable request.
