# Supplementary material for: The global prevalence of and risk factors for fear of falling among older adults: a systematic review and meta-analysis
Source: BMC Geriatr. 2024 Apr 5;24:321. doi: 10.1186/s12877-024-04882-w (PMC10998426; doi:10.1186/s12877-024-04882-w)
Supplement: Supplementary file 1 — Supplementary Material 1. [file 12877_2024_4882_MOESM1_ESM.docx]

**Supplementary Material 1 PubMed search strategies**

1#: “ Aged”[Mesh]

2#: “older” OR ”older adult” OR “older” OR “elderly” OR “the aged”

3# :1# OR 2#

4# :fear of falling

5# :“influence factors” OR “risk factors”

6# :3# AND 4# AND 5#

**Detail search strategies for all databases (**updated to September 2, 2023**)**

**1.PubMed (**847 studies**)**

1# “ Aged”[Mesh]

2# “older” OR ”older adult” OR “older” OR “elderly” OR “the aged”

3# 1# OR 2#

4# fear of falling

5# “influence factors” OR “risk factors”

6# 3# AND 4# AND 5#


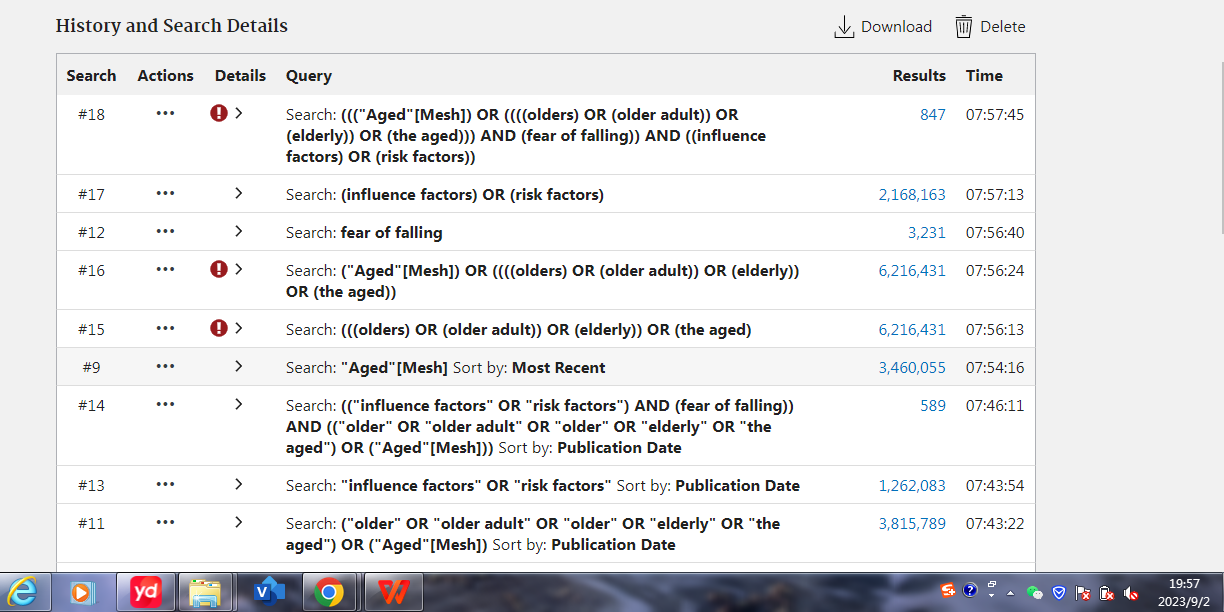


**2. Web of science (**2108 studies**)**

((TS=(fear of falling)) AND TS=(Aged OR older OR older adult OR elderly )) AND TS=(influence factors OR risk factors)

1# “ Aged”[Mesh]

2# “older” OR ”older adult” OR “older” OR “elderly” OR “the aged”

3# 1# OR 2#

4# fear of falling

5# “influence factors” OR “risk factors”

6# 3# AND 4# AND 5#


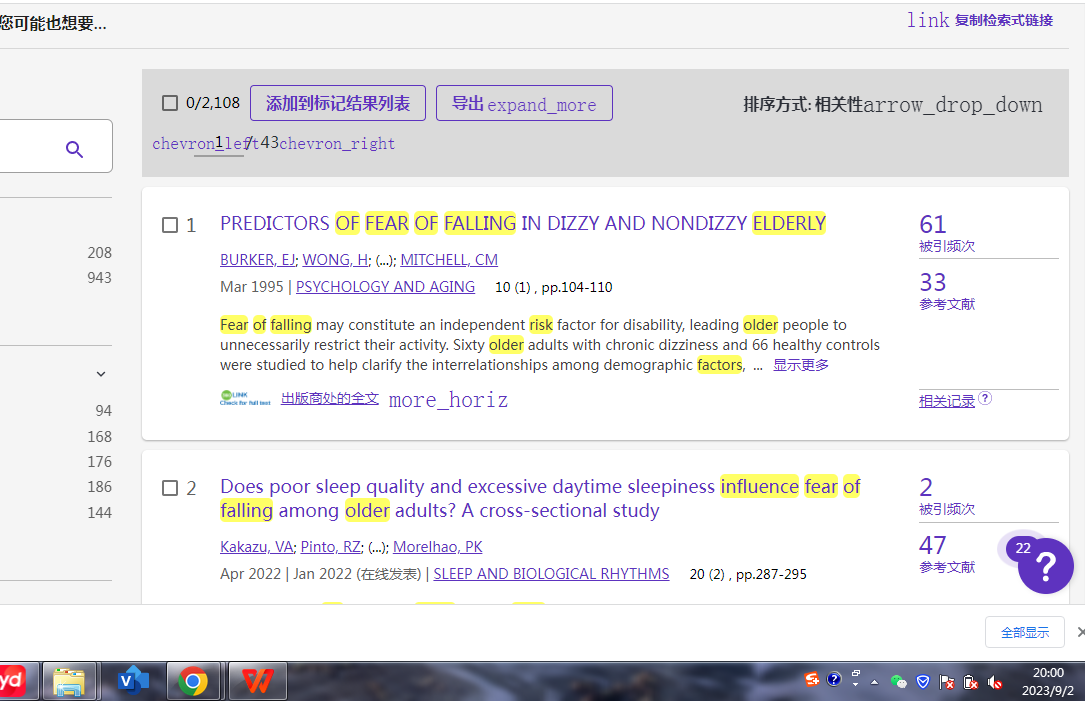


1. **Embase (346** studies**)**

**
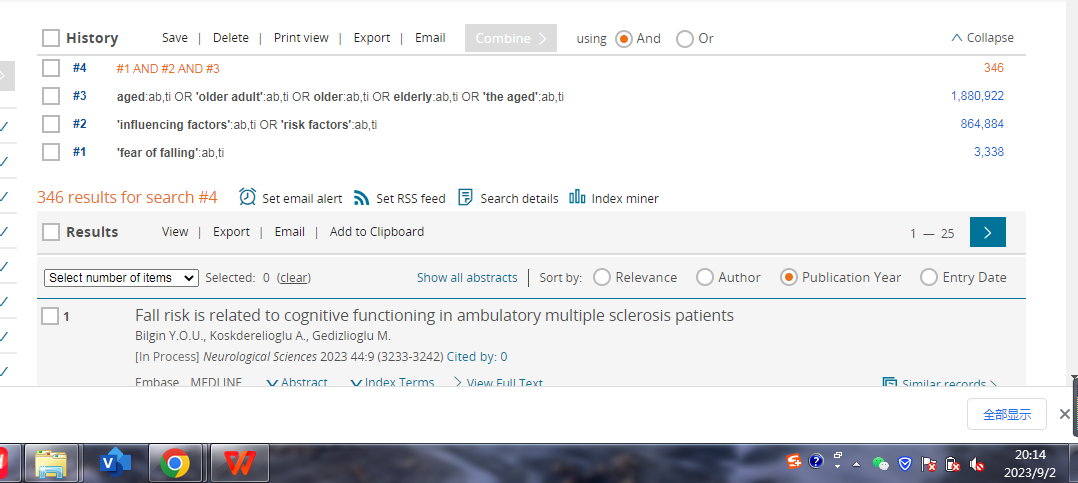
**

1. **The Cochran Library(150 studies)**

**
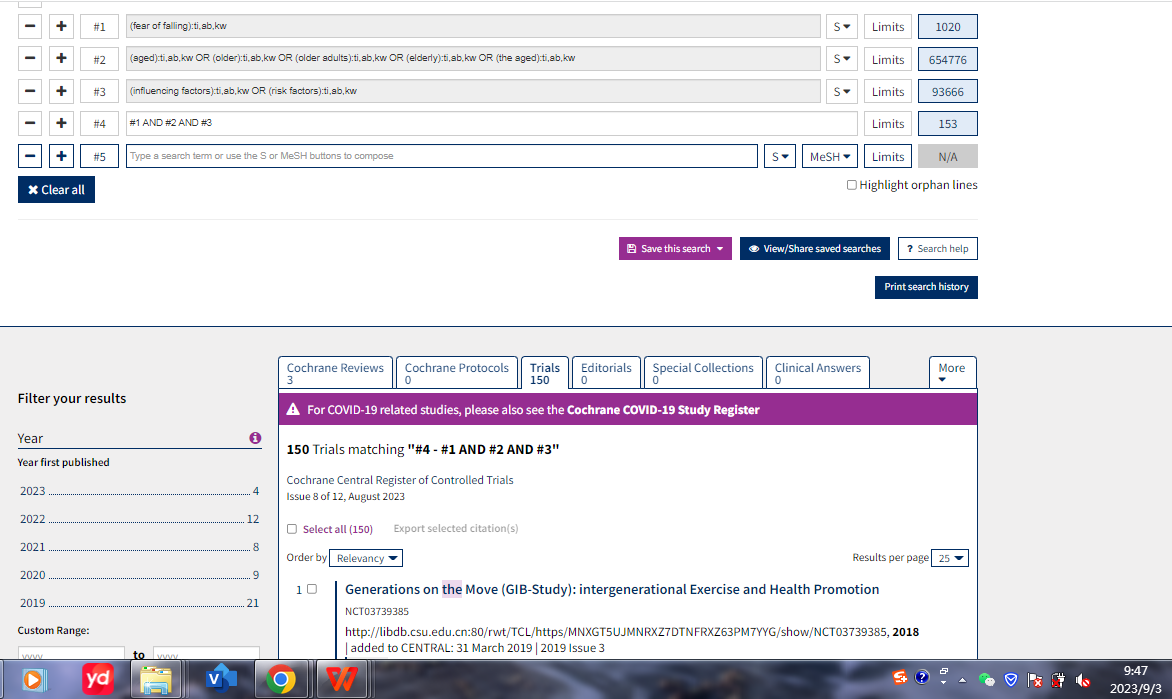
**
