# Supplementary material for: The global prevalence of and risk factors for fear of falling among older adults: a systematic review and meta-analysis
Source: BMC Geriatr. 2024 Apr 5;24:321. doi: 10.1186/s12877-024-04882-w (PMC10998426; doi:10.1186/s12877-024-04882-w)
Supplement: Supplementary file 3 — Supplementary Material 3. [file 12877_2024_4882_MOESM3_ESM.docx]

**Supplementary Material 4 The global prevalence of FOF by random effects model**

| Study | ES | [95% Conf. Interval] | % Weight |
| --- | --- | --- | --- |
| Goh 2016 | 0.728 | 0.650 - 0.806 | 0.64 |
| Damar 2021 | 0.687 | 0.625 - 0.750 | 0.65 |
| Zhang 2021 | 0.565 | 0.507 - 0.622 | 0.65 |
| Lavedán 2018 | 0.370 | 0.333 - 0.408 | 0.66 |
| Chen 2021 | 0.329 | 0.317 - 0.342 | 0.66 |
| Turhan Damar 2018 | 0.613 | 0.546 - 0.680 | 0.65 |
| Scarlett 2019 | 0.726 | 0.646 - 0.807 | 0.64 |
| Tsonga 2016 | 0.824 | 0.733 - 0.914 | 0.64 |
| James 2017 | 0.589 | 0.561 - 0.618 | 0.66 |
| Rivasi 2020 | 0.151 | 0.121 - 0.181 | 0.66 |
| Esbrí-Víctor 2017 | 0.765 | 0.704 - 0.826 | 0.65 |
| Simsek 2020 | 0.867 | 0.846 - 0.888 | 0.66 |
| Lach 2005 | 0.282 | 0.252 - 0.312 | 0.66 |
| Canever 2021 | 0.484 | 0.428 - 0.540 | 0.65 |
| Murphy 2003 | 0.268 | 0.219 - 0.317 | 0.66 |
| Oh 2017 | 0.756 | 0.747 - 0.766 | 0.66 |
| Birhanie 2021 | 0.599 | 0.555 - 0.643 | 0.66 |
| Oh-Park 2011 | 0.500 | 0.450 - 0.550 | 0.66 |
| Rochat 2010 | 0.297 | 0.266 - 0.327 | 0.66 |
| Curcio 2009 | 0.833 | 0.815 - 0.851 | 0.66 |
| Chang 2016 | 0.534 | 0.518 - 0.550 | 0.66 |
| Perez-Jara 2012 | 0.514 | 0.447 - 0.580 | 0.65 |
| Kumar 2014 | 0.192 | 0.169 - 0.216 | 0.66 |
| Singh 2020 | 0.211 | 0.200 - 0.223 | 0.66 |
| Noh 2019 | 0.644 | 0.629 - 0.658 | 0.66 |
| Kim 2013 | 0.766 | 0.757 - 0.775 | 0.66 |
| Palagyi 2017 | 0.329 | 0.278 - 0.381 | 0.65 |
| Chang 2017 | 0.534 | 0.518 - 0.550 | 0.66 |
| Boyd 2009 | 0.362 | 0.339 - 0.385 | 0.66 |
| Borges 2015 | 0.548 | 0.452 - 0.644 | 0.63 |
| Visschedijk 2013 | 0.270 | 0.183 - 0.357 | 0.64 |
| Perez-Jara 2009 | 0.500 | 0.431 - 0.569 | 0.65 |
| Visschedijk 2015 | 0.625 | 0.568 - 0.682 | 0.65 |
| Vellas 1997 | 0.320 | 0.258 - 0.381 | 0.65 |
| Park 2014 | 0.388 | 0.356 - 0.421 | 0.66 |
| Austin 2007 | 0.458 | 0.431 - 0.485 | 0.66 |
| Auais 2016 | 0.537 | 0.514 - 0.560 | 0.66 |
| Mann 2006 | 0.590 | 0.567 - 0.614 | 0.66 |
| Canever 2022 | 0.455 | 0.399 - 0.510 | 0.65 |
| Payette 2017 | 0.800 | 0.643 - 0.957 | 0.59 |
| Jellesmark 2012 | 0.576 | 0.407 - 0.744 | 0.58 |
| Uemura 2012 | 0.535 | 0.437 - 0.632 | 0.63 |
| Howland 1998 | 0.549 | 0.489 - 0.609 | 0.65 |
| Clemson 2015 | 0.145 | 0.121 - 0.169 | 0.66 |
| Zijlstra 2007 | 0.542 | 0.527 - 0.558 | 0.66 |
| Jefferis 2014 | 0.159 | 0.141 - 0.177 | 0.66 |
| Franzoni 1994 | 0.463 | 0.330 - 0.596 | 0.61 |
| Bruce 2002 | 0.340 | 0.316 - 0.364 | 0.66 |
| Rossat 2009 | 0.341 | 0.314 - 0.368 | 0.66 |
| Kressig 2001 | 0.477 | 0.420 - 0.535 | 0.65 |
| Kornfield 2017 | 0.452 | 0.406 - 0.497 | 0.66 |
| Viljanen 2012 | 0.399 | 0.353 - 0.445 | 0.66 |
| Frankenthal 2021 | 0.448 | 0.431 - 0.466 | 0.66 |
| Park 2017 | 0.757 | 0.749 - 0.765 | 0.66 |
| Mane 2014 | 0.332 | 0.274 - 0.390 | 0.65 |
| Goldberg 2022 | 0.273 | 0.180 - 0.366 | 0.64 |
| Wang 2022 | 0.885 | 0.861 - 0.909 | 0.66 |
| Zheng 2016 | 0.855 | 0.791 - 0.919 | 0.65 |
| Bertera 2008 | 0.390 | 0.374 - 0.406 | 0.66 |
| Thiamwong 2017 | 0.360 | 0.312 - 0.408 | 0.66 |
| Viljanen 2013 | 0.399 | 0.353 - 0.445 | 0.66 |
| Choi 2015 | 0.775 | 0.763 - 0.788 | 0.66 |
| Taghadosi 2018 | 0.903 | 0.875 - 0.932 | 0.66 |
| Vitorino 2019 | 0.724 | 0.676 - 0.771 | 0.66 |
| Topuz 2014 | 0.302 | 0.205 - 0.399 | 0.63 |
| Sharaf 2008 | 0.529 | 0.461 - 0.597 | 0.65 |
| Chang 2010 | 0.534 | 0.519 - 0.550 | 0.66 |
| Ivanovic 2018 | 0.558 | 0.509 - 0.606 | 0.66 |
| Kulkarni 2020 | 0.410 | 0.358 - 0.462 | 0.65 |
| Lee 2020 | 0.705 | 0.691 - 0.719 | 0.66 |
| Sitdhiraksa 2021 | 0.252 | 0.194 - 0.311 | 0.65 |
| Lee 2021 | 0.506 | 0.488 - 0.524 | 0.66 |
| Deshpande 2008 | 0.070 | 0.052 - 0.087 | 0.66 |
| Savas 2019 | 0.222 | 0.187 - 0.256 | 0.66 |
| Brodowski 2022 | 0.541 | 0.442 - 0.639 | 0.63 |
| Malini 2016 | 0.520 | 0.484 - 0.556 | 0.66 |
| Tomita 2018 | 0.365 | 0.332 - 0.397 | 0.66 |
| Lim 2011 | 0.674 | 0.642 - 0.706 | 0.66 |
| Doi 2012 | 0.622 | 0.563 - 0.681 | 0.65 |
| Murphy 2002 | 0.429 | 0.399 - 0.458 | 0.66 |
| Chu 2011 | 0.253 | 0.209 - 0.298 | 0.66 |
| Nawai 2022 | 0.562 | 0.511 - 0.613 | 0.65 |
| Drummond 2022 | 0.519 | 0.461 - 0.576 | 0.65 |
| Kurkova 2020 | 0.797 | 0.754 - 0.839 | 0.66 |
| Gupta 2022 | 0.392 | 0.339 - 0.446 | 0.65 |
| Boltz 2014 | 0.683 | 0.540 - 0.825 | 0.60 |
| Brustio 2018 | 0.684 | 0.580 - 0.789 | 0.63 |
| Hewston 2018 | 0.842 | 0.816 - 0.868 | 0.66 |
| Kakhki 2018 | 0.316 | 0.263 - 0.368 | 0.65 |
| Makino 2021 | 0.415 | 0.395 - 0.434 | 0.66 |
| Akosile 2021 | 0.553 | 0.461 - 0.644 | 0.64 |
| Nguyen 2020 | 0.881 | 0.850 - 0.913 | 0.66 |
| Vitorino 2017 | 0.665 | 0.594 - 0.736 | 0.65 |
| Teixeira 2019 | 0.789 | 0.712 - 0.866 | 0.64 |
| Liu 2021 | 0.093 | 0.073 - 0.112 | 0.66 |
| Gottschalk 2020 | 0.660 | 0.607 - 0.713 | 0.65 |
| Umegaki 2021 | 0.865 | 0.833 - 0.896 | 0.66 |
| Vo 2020 | 0.625 | 0.590 - 0.660 | 0.66 |
| Shahid 2020 | 0.759 | 0.713 - 0.805 | 0.66 |
| Liu 2015 | 0.647 | 0.603 - 0.692 | 0.66 |
| Sakurai 2021 | 0.538 | 0.466 - 0.610 | 0.65 |
| Asai 2017 | 0.223 | 0.172 - 0.274 | 0.65 |
| Du 2022 | 0.188 | 0.165 - 0.211 | 0.66 |
| Park 2022 | 0.167 | 0.153 - 0.181 | 0.66 |
| Arfken 1994 | 0.289 | 0.259 - 0.319 | 0.66 |
| Chou 2007 | 0.181 | 0.139 - 0.223 | 0.66 |
| Bahat Öztürk 2021 | 0.446 | 0.415 - 0.476 | 0.66 |
| Arani 2020 | 0.515 | 0.430 - 0.600 | 0.64 |
| Yoshikawa 2019 | 0.833 | 0.801 - 0.865 | 0.66 |
| Ren 2022 | 0.190 | 0.167 - 0.213 | 0.66 |
| Katsumata 2011 | 0.293 | 0.258 - 0.328 | 0.66 |
| Friedman 2002 | 0.208 | 0.191 - 0.224 | 0.66 |
| Löppönen 2022 | 0.718 | 0.678 - 0.758 | 0.66 |
| Lach 2020 | 0.507 | 0.441 - 0.572 | 0.65 |
| van Haastregt 2008 | 0.448 | 0.406 - 0.490 | 0.66 |
| Schroeder 2022 | 0.568 | 0.522 - 0.615 | 0.66 |
| Sakurai 2017 | 0.504 | 0.414 - 0.595 | 0.64 |
| Aburub 2020 | 0.233 | 0.189 - 0.277 | 0.66 |
| Gagnon 2005 | 0.457 | 0.362 - 0.552 | 0.63 |
| Visschedijk-2014 | 0.500 | 0.402 - 0.598 | 0.63 |
| Jaatinen 2022 | 0.493 | 0.461 - 0.526 | 0.66 |
| Martínez-Arnau 2021 | 0.511 | 0.446 - 0.576 | 0.65 |
| De Roza 2022 | 0.608 | 0.558 - 0.659 | 0.65 |
| Pohl 2015 | 0.461 | 0.396 - 0.525 | 0.65 |
| Trevisan 2020 | 0.461 | 0.442 - 0.480 | 0.66 |
| Choi 2017 | 0.385 | 0.360 - 0.409 | 0.66 |
| Merchant 2020 | 0.692 | 0.651 - 0.732 | 0.66 |
| Asai 2022 | 0.540 | 0.497 - 0.582 | 0.66 |
| Yang 2020 | 0.532 | 0.389 - 0.675 | 0.60 |
| Freiberger 2022 | 0.787 | 0.746 - 0.827 | 0.66 |
| Peterson 1999 | 0.700 | 0.645 - 0.755 | 0.65 |
| Sawa 2023 | 0.489 | 0.479 - 0.499 | 0.66 |
| You 2023 | 0.316 | 0.306 - 0.326 | 0.66 |
| Garbin 2023 | 0.113 | 0.089 - 0.137 | 0.66 |
| Liu 2023 | 0.286 | 0.275 - 0.298 | 0.66 |
| Scheffers-Barnhoorn 2023 | 0.572 | 0.526 - 0.618 | 0.66 |
| Prado 2023 | 0.455 | 0.399 - 0.510 | 0.65 |
| Zhang 2023 | 0.553 | 0.511 - 0.595 | 0.66 |
| chu 2023 | 0.232 | 0.175 - 0.289 | 0.65 |
| Canever 2021 | 0.455 | 0.399 - 0.510 | 0.65 |
| Wang 2022 | 0.885 | 0.861 - 0.909 | 0.66 |
| Freiberger 2022 | 0.787 | 0.746 - 0.827 | 0.66 |
| Siefkas 2022 | 0.243 | 0.227 - 0.259 | 0.66 |
| Korenhof 2023 | 0.499 | 0.478 - 0.520 | 0.66 |
| Garbin 2023 | 0.376 | 0.363 - 0.390 | 0.66 |
| Dos Santos 2023 | 0.439 | 0.391 - 0.487 | 0.66 |
| DiGuiseppi 2022 | 0.186 | 0.172 - 0.200 | 0.66 |
| Badrasawi 2022 | 0.490 | 0.421 - 0.559 | 0.65 |
| McKay 2022 | 0.302 | 0.244 - 0.359 | 0.65 |
| Luo 2022 | 0.292 | 0.281 - 0.303 | 0.66 |
| Shiratsuchi 2022 | 0.354 | 0.345 - 0.364 | 0.66 |
| Turhan Damar 2022 | 0.888 | 0.857 - 0.918 | 0.66 |
| Dhar 2022 | 0.420 | 0.386 - 0.454 | 0.66 |
| D+L pooled ES | 0.496 | 0.459 - 0.532 | 100.00 |

Note: Heterogeneity chi-squared = 50648.15 (d.f. = 152) p = 0.000, I-squared (variation in ES attributable to heterogeneity) = 99.7%, Estimate of between-study variance Tau-squared = 0.0514, Test of ES=0 : z= 26.84 p = 0.000.
